# The impact of community-based palliative care on acute hospital use in the last year of life is modified by time to death, age and underlying cause of death. A population-based retrospective cohort study

**DOI:** 10.1371/journal.pone.0185275

**Published:** 2017-09-21

**Authors:** Katrina Spilsbury, Lorna Rosenwax, Glenn Arendts, James B. Semmens

**Affiliations:** 1 Centre for Population Health Research, Faculty of Health Sciences, Curtin University, Perth, Australia; 2 School of Occupational Therapy and Social Work, Faculty of Health Sciences, Curtin University, Perth, Australia; 3 Centre for Clinical Research in Emergency Medicine, Harry Perkins Institute of Medical Research, The University of Western Australia, Perth, Australia; 4 Department of Emergency Medicine, Fiona Stanley Hospital, Perth, Australia; 5 Institute for Health Research, The University of Notre Dame, Fremantle, Australia; Cardiff University, UNITED KINGDOM

## Abstract

**Objective:**

Community-based palliative care is known to be associated with reduced acute care health service use. Our objective was to investigate how reduced acute care hospital use in the last year of life varied temporally and by patient factors.

**Methods:**

A retrospective cohort study of the last year of life of 12,763 Western Australians who died from cancer or one of seven non-cancer conditions. Outcome measures were rates of hospital admissions and mean length of hospital stays. Multivariate analyses involved time-to-event and population averaged log-link gamma models.

**Results:**

There were 28,939 acute care overnight hospital admissions recorded in the last year of life, an average of 2.3 (SD 2.2) per decedent and a mean length of stay of 9.2 (SD 10.3) days. Overall, the rate of hospital admissions was reduced 34% (95%CI 1–66) and the mean length of stay reduced 6% (95%CI 2–10) during periods of time decedents received community-based palliative care compared to periods of time not receiving this care. Decedents aged <70 years receiving community-based palliative care showed a reduced rate of hospital admission around five months before death, whereas for older decedents the reduction in hospital admissions was apparent a year before death. All decedents who were receiving community-based palliative care tended towards shorter hospital stays in the last month of life. Decedents with neoplasms had a mean length of stay three weeks prior to death while not receiving community-based palliative care of 9.6 (95%CI 9.3–9.9) days compared to 8.2 (95% CI 7.9–8.7) days when receiving community-based palliative care.

**Conclusion:**

Rates of hospital admission during periods of receiving community-based palliative care were reduced with benefits evident five months before death and even earlier for older decedents. The mean length of hospital stay was also reduced while receiving community-based palliative care, mostly in the last month of life.

## Introduction

Community-based palliative care, sometimes called home-based palliative care, has consistently been shown to be associated with reduced acute care health service use and improved patient wellbeing in a variety of different settings.[1–6] Historically palliative care research has focussed on patients with advanced cancers, however it is now clear that specialist palliative care, home-based or hospital-based, also benefits people with non-cancer chronic and life-limiting conditions. [7–10] Despite this, access to specialist palliative care for non-cancer conditions is generally considered sub-optimal [11–14] although there are signs that this is improving.[14, 15]

The effectiveness of community-based palliative care in reducing use of acute care health services is not uniform and has been shown to vary by type of service, patient and temporal factors. We reported previously that community-based palliative care for cancer and four non-cancer conditions was associated with a greater reduction in emergency department visits in the last year of life in patients that were older, had a partner at time of death and lived in areas of higher socioeconomic status. [5] An Italian study reported that the intensity of specialist home-based palliative care delivered to a cohort of patients with non-cancer chronic disease was associated with reduced hospital stays. [7] A large Canadian study reported a reduction in hospitalisations in the week following home nursing with end-of-life intent in a cohort of cancer patients followed for the last six months of life. [16]

The timing of palliative care provision is of interest with evidence that early palliative care in oncology is beneficial for patient well-being, less acute care health service use, and in the case of lung cancer, improved survival.[17–19] Recommendation of palliative care at the time of diagnosis for patients with advanced cancer is now being integrated in clinical guidelines. [20] However, it is less clear what defines early community-based palliative care in non-cancer conditions and whether it is associated with reduced acute care health services. A study of hospital outpatient early palliative care in end-stage liver disease found improvements in patient mood scores[21] and the effect of early hospital-based palliative care intervention in chronic obstructive pulmonary disease (COPD) is being investigated in a randomised controlled trial setting. [22] There have been few studies that have directly compared rates of hospital use in the last year of life between cancer and individual non-cancer conditions amenable to palliative care and how this is modified by accessing community-based palliative care in the last year of life.

The aim of this study was to investigate whether community-based palliative care was associated with reduced acute care hospital use in the last year of life in a population-based cohort of patients who died with cancer and seven other non-cancer conditions. We were particularly interested in exploring how the benefit of community-based palliative care varied temporally and with patient sociodemographic and health-related factors.

## Methods

This was a retrospective cohort study that used a linked de-identified extraction of death records, hospital records and community-based palliative care records of people who died in Western Australia (WA) from 1 January 2009 to 31 December 2010. All hospital records and community-based care records pertaining to the last year of life plus a one year look back period for identifying comorbid conditions were extracted. Data was obtained from the Data Linkage Branch at the WA Department of Health and ethical approval to conduct this study was provided by Human Research Ethics Committees at the WA Department of Health and Curtin University.

### Cohort selection

The overall end-of-life cohort has been described previously. [15] Briefly, it consisted of 12, 817 people who died during the two year study period in Western Australia and who had mention on Part I of their death certificate of one or more of the ten disease conditions considered amenable for palliative care as defined by Rosenwax et al.[23] The conditions of interest were cancer, heart failure, renal failure, liver failure, chronic obstructive pulmonary disease (COPD), Alzheimer's disease, motor neurone disease, Parkinson's disease, Huntington's disease and HIV/AIDS. When a decedent had more than one of the ten conditions of interest on Part 1 of their death certificate, then the most antecedent condition was assigned as the principal condition.

### Specialist palliative care

Community-based palliative care was defined as the palliative care service provided by the not-for-profit organisation, Silver Chain WA.[24] Silver Chain WA provided the majority of community-based palliative care in WA during the study period, although the most intensive service was restricted to the major metropolitan areas. Palliative care during the study period was generally provided after referral from community or hospital based medical practitioners when the client had progressive and terminal disease. Access was not restricted by diagnosis or predicted time to death. A multidisciplinary team of clinical nurses, clinicians, carers, social workers, pastoral care and volunteers provided at-home nursing care and practical support, symptom management, counselling, respite options and referral to other community and government services. A palliative nurse consultancy service was available to public/private hospitals and residential care facilities where client care is managed by a registered nurse 24 hours each day. This includes advice, assessment, staff education and telephone follow-up to meet the needs of a specific client. A palliative rural telephone advisory service provided specialist advice to local rural service providers 24 hours per day.

### Decedent social, demographic and health variables

Marital status was classified as partnered (married or de-facto) or not/unknown. Decedents’ addresses at time of hospital admissions and at death were used to classify type of residence as private, residential aged care facility (RACF) or other care facility. Address was also use to assign accessibility categories based on the Accessibility/Remoteness Index of Australia (ARIA+) that takes into account road distance measurements to the nearest Service Centres and population size[25]. Private hospital insurance was defined as any record of a hospital stay as a private hospital patient during the last year of life.

Comordity was estimated from the first 21 hospital record diagnostic fields containing International Classification of Disease 10 Australian modification (ICD-10-AM codes) spanning the last two years of life by summing the number of the 31 medical conditions identified by Elixhauser[26] based on algorithms created by Quan.[27] If the principal underlying causes of death matched any conditions included in the Elixhauser comorbid conditions, it was excluded from the calculation of the number of comorbid condtions. Comorbidity for decedents without any hospital admissions in the last two years of life was estimated from Australian Bureau of Statistics coded causes of death obtained from the death certificate.

### Hospital stays

Hospital data were provided at the level of individual episodes of care. An episode of care is defined as period of admitted patient care characterised by only one care type. A hospital stay can be made up a single episode of care (e.g. one episode of acute care) or multiple episodes of care (e.g. a period of acute care followed by a period of rehabilitation care). For this study a hospital stay was defined as continuous occupancy of an inpatient bed. Most hospital stays involved a single care type (e.g. acute care). When multiple types of care were present, they were brought together into a single hospital stay including statistical discharges (e.g. change of care type from acute care to palliative) or transfer to another hospital. Short episodes of care nested within longer episodes of care (e.g. a patient receiving dialysis while admitted for a different condition) were similarly included within the same hospital stay. An acute care hospital admission was defined as a hospital stay where the initial type of care was acute. Acute care stays nested within other non-acute hospital stays were not considered an acute care admission in this study. Acute care hospital admissions were further categorised as day stay and overnight stay admissions.

### Statistical methods

Data were structured so that each decedent had 365 observations representing every day of their last year of life. Each day was assigned as being a day of a hospital stay (and type) or not. Similarly, each day was assigned as receiving community-based palliative care or not based on each decedent’s Silver Chain palliative care service enrolment and disenrollment dates. It was possible for patients to have multiple periods of palliative care enrolment if their symptoms were of a relapsing and remitting nature. Dates often overlapped because a day coded as a hospital stay could also be a community-based palliative care day if it fell between Silver Chain service dates.

Factors associated with the relative rate (hazard) of overnight acute care hospital admissions during the last year of life were investigated using flexible parametric multiple failure proportional hazards (Royston-Parmar) models [28] with decedents excluded from the risk pool during periods of hospital stays. A decedent’s history of hospital admissions in last year of life and community-based palliative care status were entered as time-varying covariates into the model. Likelihood ratio tests were used to identify variables with time-dependent effects and the best fitting spline functions were identified by minimising the Akaike Information Criteria. In addition to being a time-varying covariate, community-based palliative care status also showed time-dependent (non-proportional) effects which were characterised in the final model as a seven-knot spline function.

Factors associated with the mean length of hospital stays were investigated using population averaged generalised estimating equations assuming a gamma distribution and log link function with an exchangeable correlation structure and robust variance estimators. This approach was conditional upon having an overnight stay and used to account for the within-person correlation of hospital length of stay and skewness of the data. Predictions of the mean length of hospital stay were derived from the final adjusted regression model.

Variables tested for parsimonious inclusion in all multivariate models were underlying cause of death, age at death, sex, private health insurance status, ARIA+ accessibility, partner status, number of comorbid conditions and type of residence. Interaction terms of covariates with the main variable of interest, community-based palliative care status, were tested for inclusion. Fractional polynomial transformation of continuous variables were used when model fit was improved. The Sidak adjustment to p-values was applied when testing multiple hypotheses simultaneously. All data management and analysis was conducted using Stata 14 (College Station, TX).

## Results

There were 12,817 decedents in the original palliative care cohort with one or more of the ten minimal disease conditions considered amenable to palliative care listed on Part I of their death certificate. Due to low numbers, 17 decedents with Huntington’s disease or HIV/AIDs were excluded as were 37 decedents with missing residential address information. There remained 12,763 decedents in the study cohort. Neoplasms were the most common underlying cause of death (57.9%) followed by heart failure (15.8%), renal failure (8.9%), COPD (8.5%), Alzheimer’s dementia (4.7%), liver failure (1.6%), Parkinson’s disease (1.4%) and motor neurone disease (1.1%). Almost one third (n = 3884, 30.4%) of decedents accessed community-based palliative care at least once in the last year of life.

### Hospital use in last year of life

In the last year of life, the majority of the cohort (n = 11,494; 90.1%) were admitted to hospital at least once, generating 83,796 hospital admissions and accounting for 9.9% (n = 459,033 days) of days in the last year of life. Of all days spent in hospital by the cohort in their last year of life, 74.6% of the days were for acute care. Of the 80,228 acute care admissions, 28,939 (36.1%) were admissions involving overnight acute care stays and 54,857 (63.9%) were as a hospital day stay. As most day stay acute admissions were planned admissions for chemotherapy and renal dialysis, only overnight acute care admissions were investigated further.

Decedents had an average of 2.3 (SD 2.2) overnight acute care hospital stays in the last year of life, ranging from zero stays for 2,178 (17.1%) decedents to 28 overnight acute care stays for one decedent. In general, higher average numbers of acute care overnight hospital stays were observed in in decedents who were younger, male, partnered, had private health insurance, more comorbid conditions, did not live in a RACF and died from neoplasms and liver failure (Table 1). The mean length of stay for overnight acute care hospital stays for the cohort as a whole was 9.2 (SD 10.3) days and median length of stay of 6 (IQR 3–11) days. Decedents who were older at time of death, had multiple comorbid conditions and died from renal failure, liver failure, or Alzheimer’s disease tended to have longer hospital stays (Table 1).

**Table 1:** The mean and median number of overnight acute care hospital stays and the mean and median length of stay in the last year of life by decedent factors (n = 12,763).

Decedent factors	N	No. overnight stays				Length of stay (days)			
		Mean	SD	Med	IQR	Mean	SD	Med	IQR
**Age at death**									
<60	1640	3.0	2.7	2	1–4	8.7	10.9	5	3–10
60–69	1994	2.6	2.3	2	1–4	8.9	9.8	6	3–11
70–79	3200	2.4	2.1	2	1–3	9.3	10.1	6	3–11
80–89	4208	2.0	2.0	2	1–3	9.6	10.2	6	3–12
90+	1721	1.5	1.7	1	0–2	9.4	10.1	6	3–12
**Sex**									
Male	6910	2.4	2.2	2	1–3	9.1	10.2	6	3–11
Female	5853	2.1	2.1	2	1–3	9.4	10.3	6	3–12
**Partnered at death**									
No	6825	2.1	2.1	2	1–3	9.7	10.9	6	3–12
Yes	5938	2.5	2.2	2	1–3	8.8	9.6	6	3–11
**Location of residence**									
Major cities	8767	2.3	2.1	2	1–3	9.5	10.7	6	3–12
Inner regional	2121	2.2	2.1	2	1–3	8.6	8.9	6	3–11
Outer regional	1216	2.3	2.2	2	1–3	8.6	9.6	5	3–10
Remote	425	2.4	2.3	2	1–3	8.5	10.3	5	3–10
Very remote	234	2.8	2.9	2	1–4	7.6	9.1	5	3–9
**Private health insurance**									
No	8227	1.9	2.0	1	1–3	9.2	10.0	6	3–11
Yes	4536	2.9	2.4	2	1–4	9.1	10.6	6	3–11
**Type of residence**									
Private residence	9754	2.5	2.2	2	1–3	9.2	10.3	6	3–11
RACF	2711	1.4	1.7	1	0–2	9.1	9.8	6	3–11
Other	298	1.8	2.0	1	0–3	9.1	9.7	6	3–11
**Underlying cause of death**									
Neoplasms	7391	2.5	2.2	2	1–3	9.0	10.0	6	3–11
Heart failure	2017	2.1	2.1	2	1–3	9.4	9.7	6	3–12
Renal failure	1138	2.3	2.0	2	1–3	9.9	11.9	6	3–12
COPD	1089	2.3	2.2	2	1–3	9.7	10.7	7	4–12
Alzheimer’s	605	0.6	0.9	0	0–1	9.9	10.1	7	4–12
Liver failure	206	3.1	3.0	2	1–4	9.6	11.1	6	3–12
Motor neurone disease	136	1.5	1.5	1	0.5–2	8.1	7.7	5	3–11
Parkinson’s	181	1.0	1.3	1	0–2	9.4	8.8	7	4–12
**No. comorbid conditions**									
None	2852	1.1	1.5	1	0–2	8.0	8.4	5	3–10
One	2790	2.2	1.9	2	1–3	8.3	9.3	5	3–10
Two	2192	2.4	1.9	2	1–3	9.2	9.9	6	3–11
Three	1654	2.7	2.2	2	1–3	9.3	9.8	6	3–11
Four or more	3275	3.3	2.4	3	2–4	10.0	11.5	6	3–12

RACF, residential aged care facility; COPD, chronic obstructive pulmonary disease; IQR, interquartile range; SD, standard deviation; Med, Median

### Rate of overnight acute care hospital admissions

**Fig 1:**
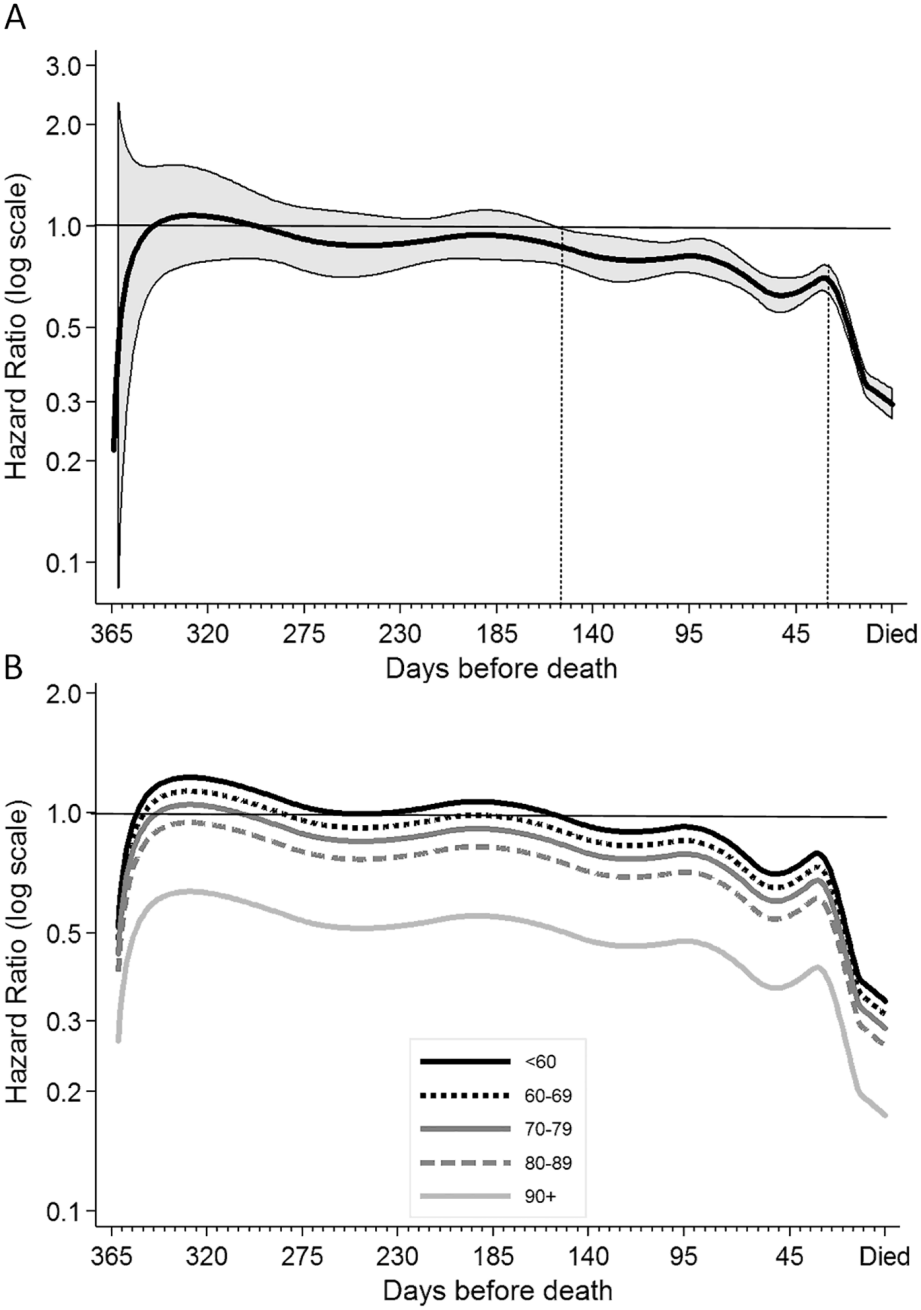
Adjusted relative hazard (rate) of overnight acute care hospital admissions during periods of time receiving community-based palliative care over the last year of life compared to periods of time not receiving community-based palliative care. A) Hazard rate (HR) averaged over all age groups (thick black line) with 95% confidence interval (CI) indicated by shading. The time at which the upper 95% CI drops below the referent line and the time at which the HR reduction becomes more rapid are indicated by dotted grey lines. B) HR (without 95%CI shading) for each age group as indicated by legend. Referent line shown at HR of 1 represents periods of time not receiving community-based palliative care.

Multivariate analyses estimated that the rate of overnight acute care hospital admissions was reduced 34% (HR 0.66, 95%CI 0.44–0.99) during periods of time decedents received community-based palliative care compared to periods not receiving palliative care when averaged over the last year of life and adjusting for other covariates and closeness to time of death. However, the strength of this association varied over the last year of life. In addition, a significant interaction between the time-dependent community-based palliative care status variable and age at death was observed. This means that the reduced rate of hospital admissions associated with receiving community-based palliative care was not constant over time and varied by decedent age and is best represented graphically (Fig 1).

In the first seven months of the year for the cohort as a whole and ignoring the effect of age, there was very little difference in the rate of acute care overnight hospital admissions whether decedents were receiving community-based palliative care or not (Fig 1A). At around five months (150 days) before death there was a steady decline in the relative reduction of the rate of acute care hospital stays during periods of time receiving community based palliative care. At around 30 days before death, the relative rate decreased more rapidly, reaching a 70% (95% CI 67–73) reduced rate of hospital admissions around the time of death.

When the effect of age on community-based palliative care was averaged over the last year of life, the positive association of community-based palliative care with a reduced rate of hospital admissions was only observed in people aged 80 years and over (Table 2). However, by taking the time-dependent pattern of the association of community-based palliative care with rate of hospital admissions into account, a reduced rate of overnight acute care hospital admissions while receiving community-based palliative care was observed in all age groups but it occurred at different times (Fig 1B). Decedents aged less than 70 years began to show a reduced rate of acute care admissions while receiving community-based palliative care around three to six months before death, whereas people aged 80 years and older receiving community-based palliative care showed reduced rates of acute care hospital admissions over the whole last year of life.

**Table 2:** Decedent health and sociodemographic factors associated with variation in the rate of overnight acute care admissions in the last year of life estimated from a multivariate time-to-event model with time varying covariates and time-dependent effects (N = 12,763 decedents).

Decedent factors^†^		HR	95%CI	p-value
2-way interaction*				
Community-based palliative care	<60	0.83	0.55–1.25	0.380
(yes vs. no) within each age group	60–69	0.77	0.51–1.16	0.210
and averaged over the last year of life	70–79	0.71	0.47–1.07	0.101
(see Fig 1B for how these varied by time)	80–89	0.64	0.43–0.97	0.034
**Main effects**				
**Underlying cause of death**	Neoplasms	1.0	ref	-
	Heart failure	0.84	0.81–0.87	<0.001
	Renal failure	0.82	0.78–0.86	<0.001
	COPD	0.96	0.92–1.00	0.073
	Alzheimer’s	0.52	0.47–0.58	<0.001
	Liver failure	0.95	0.87–1.03	0.178
	Motor neurone disease	0.63	0.55–0.72	<0.001
	Parkinson’s	0.59	0.51–0.68	<0.001
**Accessibility index**	Major cities	1.0	ref	-
	Inner regional	0.97	0.94–1.00	0.064
	Outer regional	1.02	0.98–1.07	0.228
	Remote	1.06	1.00–1.13	0.068
	Very remote	1.09	1.01–1.18	0.031
**Private health insurance**	No	1.0	ref	-
	Yes	1.30	1.27–1.34	<0.001
**Residence at death**	Private residence	1.0	ref	-
	RACF	0.60	0.58–0.63	<0.001
	Other	0.83	0.76–0.89	<0.001
**Number of comorbid conditions**	None	1.0	ref	-
	One	1.43	1.37–1.49	<0.001
	Two	1.71	1.64–1.79	<0.001
	Three	2.04	1.95–2.13	<0.001
	Four or more	2.64	2.54–2.74	<0.001

RACF, residential aged care facility; COPD, chronic obstructive pulmonary disease; HR, hazard ratio; CI, confidence interval.

*HR from a 2-way interaction term between community-based palliative care and age group averaged over the last year of life. As community-based palliative care was time varying, the effect of age group over time is visualised in Fig 1B.

^†^Model also included the time varying covariate indicating the number of prior hospital admissions in the last year of life (n ranged from 1 to 6 or more) which was entered into the model as (-2, 3) fractional polynomial.

Decedents with heart failure, renal failure, Alzheimer’s disease, motor neurone disease and Parkinson’s disease all had lower rates of acute care admissions relative to decedents with cancers. There was no difference in rates of admission between decedents with cancer, liver failure or COPD. Other factors independently associated with increased rates of overnight acute care admissions were private health insurance, living in less accessible areas and a private residence and increasing comorbidity (Table 2). The magnitude of the association of community-based palliative care with reduced rates of hospital admissions was the same regardless of underlying cause of death (interaction test p-value = 0.625).

### Length of acute care overnight hospital stays

**Fig 2:**
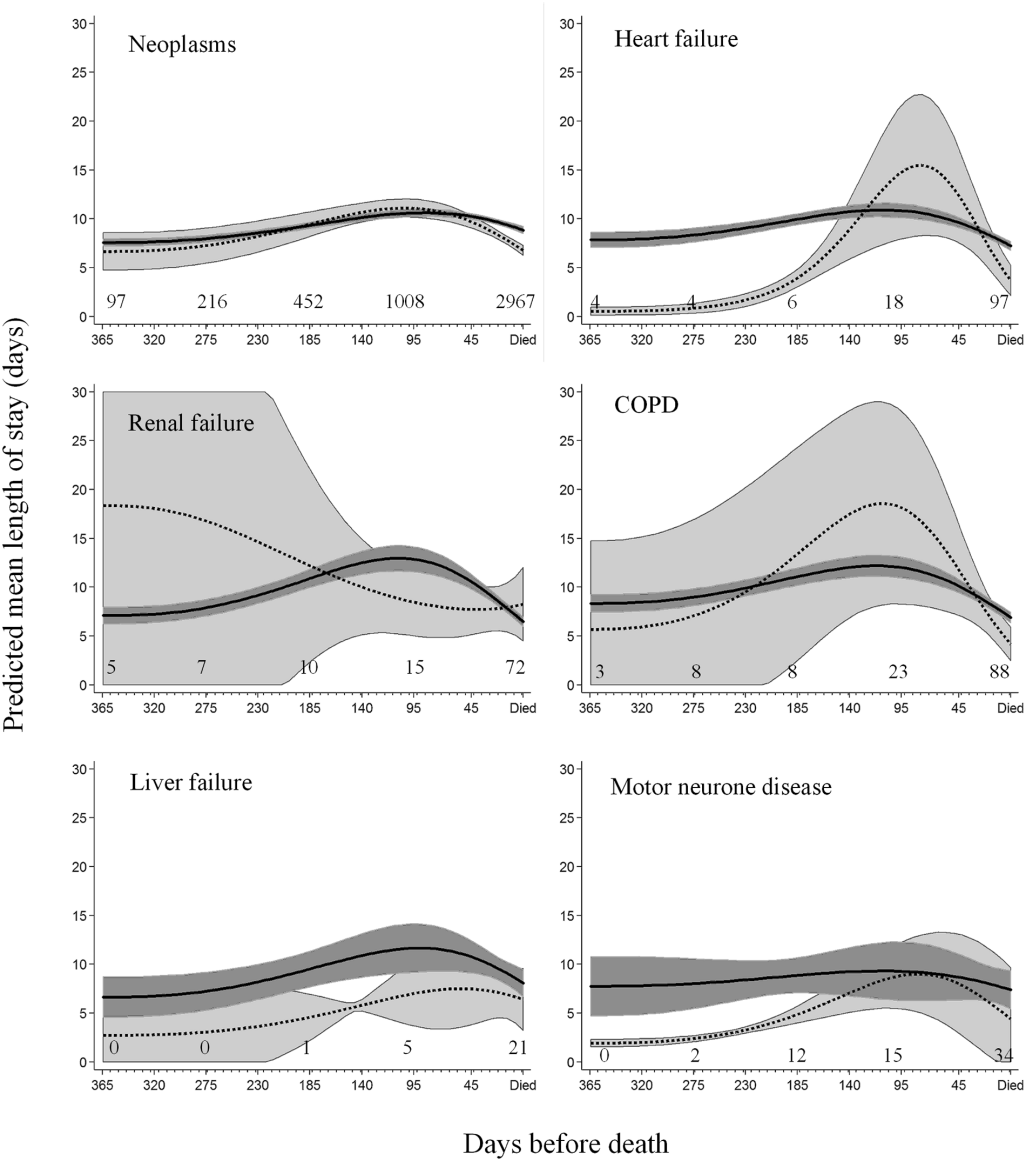
Predicted mean length of stay for overnight acute care hospital admissions for each underlying cause of death by periods of time receiving and not receiving community-based palliative care. Periods of time receiving community-based palliative care (dashed line) and periods of time not receiving community-based palliative care (solid line) over the last year of life. Estimates predicted from regression model depicted in Table 3 plus the inclusion of significant interaction terms between community-based palliative care, closeness to time of death and cause of death. 95% confidence intervals around mean lengths of stay are indicated by shading. Closeness to death in days was entered in the model as the two terms created from a (3, 3) fractional polynomial transformation. Decedents with Alzheimer’s and Parkinson’s disease had insufficient data to make a meaningful plot. The number of decedents enrolled in community-based palliative care at 365, 270, 180, 90 and 1 days before death are indicated on the graphs.

The average predicted mean length of stay during acute care admissions over the last year of life decreased 6% (HR 0.94; 95% CI 0.90–0.98) during periods when decedents were receiving community-based palliative care compared to not receiving community-based palliative care and after taking the closeness to death and other covariates into account. This equated to a reduction of 0.5 (95% CI 0.1–0.9) mean days from 9.4 days to 8.9 days per overnight acute care hospital admission. However, this varied significantly by both the principal underlying causes of death and closeness to time of death as evidenced by a significant three way interaction between community-based palliative care, underlying cause of death and closeness to death in the regression model, depicted graphically in Fig 2. The number of decedents who were enrolled in community-based palliative care at 365, 270, 180, 90 and 1 day(s) before death are indicated on the figure for each of the life-limiting conditions. The proportion of decedents accessing community-based palliative care 365 days before death relative to the numbers on the day before death ranged from 3% for neoplasms and COPD up to 7% for renal failure. There were so few decedents with Alzheimer’s disease and Parkinson’s disease that they were excluded from Fig 2.

In general, the mean length of hospital stays tended to increase with closeness to time of death, reaching a maximum average length of stay around three months prior to death, then declining until time of death. All decedents who were receiving community-based palliative care tended towards shorter hospital stays in the last month of life. Evidence for this was greatest for decedents with neoplasms with mean length of stay three weeks prior to death while not receiving community-based palliative care of 9.6 (95%CI 9.3–9.9) days compared to 8.2 (95% CI 7.9–8.7) days when receiving community-based palliative care.

Decedents with heart failure, liver failure or motor neurone disease receiving community-based palliative care more than three months before death tended to have shorter hospital stays compared to people not receiving community-based palliative care. However, decedents with neoplasms receiving community-based palliative care more than three months before death were more numerous and there was no evidence of a similar trend of shorter hospital stays. Decedents with renal failure showed very little difference in mean length of acute care hospital stays whether or not they were receiving community-based palliative care. Other covariates associated with a reduction in predicted mean length of stays were younger age, living in a RACF, having a partner, less comorbid conditions, and living outside of major metropolitan areas (Table 3).

**Table 3:** Decedent health and sociodemographic factors associated with relative variation in the mean length of overnight acute care admissions in the last year of life for decedents with at least one overnight acute care hospital stay in the last year of life (N = 28,939 admissions and 10,585 decedents).

Decedent factors		IRR	95%CI	p-value
**3-way interaction of CBPC x underlying** **cause of death x closeness to time of death**			See Fig 2	
**Age at death**	<60	1	ref	-
	60–69	1.03	0.98–1.09	0.193
	70–79	1.06	1.01–1.11	0.019
	80–89	1.09	1.03–1.14	0.001
	90+	1.08	1.01–1.15	0.018
**Accessibility Index**	Major cities	1.0	ref	-
	Inner regional	0.90	0.87–0.94	<0.001
	Outer regional	0.90	0.86–0.95	<0.001
	Remote	0.90	0.83–0.98	0.013
	Very remote	0.77	0.70–0.85	<0.001
**Partnered at death**	No	1	ref	-
	Yes	0.90	0.88–0.93	<0.001
**Residence at death**	Private residence	1	ref	-
	RACF	0.90	0.86–0.94	<0.001
	Other care facilities	0.92	0.84–1.00	0.062
**Number of comorbid conditions**	None	1	ref	-
	One	1.06	1.01–1.11	0.017
	Two	1.17	1.11–1.22	<0.001
	Three	1.17	1.12–1.23	<0.001
	Four or more	1.27	1.21–1.32	<0.001

CBPC, community-based palliative care; RACF, residential aged care facility; IRR, incidence rate ratio; CI, confidence interval.

## Discussion

We report that periods of time receiving community-based palliative care were associated with a reduced rate of overnight hospital admissions for acute care in the last year of life. This finding supports other studies that report home-based palliative hospice care being associated with reduced hospitalizations. [1, 4, 6, 29] We observed that this association of community-based palliative care with reduced hospital admissions was consistent regardless of the underlying cause of death, be it cancer, COPD, organ failure or neurological conditions. Others have investigated the impact of home based palliative care on some non-cancer conditions. A New Zealand study showed a one-year hospice programme was associated with a reduction in hospital admissions for COPD patients.[8] An Italian study reported home-based care reduced prolonged stays in hospital in the last month of life for non-cancer deaths, however decedents older than 85 years, living in a nursing home or who were not hospitalised in the last year of life were excluded, thus limiting the generalisation of findings.[7] A US based study found less hospital use in the last months of life in patients with cancer, COPD, dementia and heart failure who had received structured home-based palliative care. [2]

This association of community-based palliative care with a reduced rate of acute care overnight hospital stays was not constant over the last year of life. On average, the beneficial association became apparent around five months before death, with an even greater impact in the last month of life. This trend was consistent across all principal underlying causes of death. A similar trend was reported in a Canadian population-based study of cancer patients where reduced rates of hospitalisation in the week following a week of end-of-life home nursing were observed at six months and a greater reduction at one month before death, although the investigators did not report on earlier time periods. [16]

While there appeared to be no reduction in rate of hospital admissions with community-based palliative care accessed earlier than five months from death for the cohort as a whole, this was not the case in patients 70 years and over, who as a group benefited more than the younger patients. Yet, we and others have previously reported that older persons are less likely to receive specialist palliative care in general [15, 30, 31], let alone early palliative care.

The benefits of early integration of palliative care into the oncology setting are well documented[32, 33] and have resulted in updated clinical guidelines recommending patients with advanced cancer receiving palliative care early and concurrent with active treatment. [20] Studies have reported early referral to palliative care has advantages such as better pain management, quality of life, less aggressive end-of-life care and, in advanced lung cancer, prolonged survival.[32, 33] Our results suggest that community-based palliative care accessed earlier than three months before death may result in shorter hospital stays for those with heart failure, liver failure and motor neurone disease, however a larger studies are needed to confirm this due to low enrolment in community-based palliative care. Future studies that take date of diagnosis and severity of disease condition into account are also required in order to establish a definition of early palliative care specific to each non-cancer life limiting condition.

Community-based palliative care was associated with a 6% decrease in the average length of stay for overnight acute care hospital admissions over the last year of life. Most of this decrease was evident only in the last months of life, particularly for decedents with cancer who made up the majority of the cohort. For non-cancer conditions, evidence of this decrease in mean lengths of stay was less consistent. An Italian study reported home-based palliative care in non-cancer conditions was associated with less prolonged stays in hospital[7] and a recent USA-based study reported less hospital bed days for patients with COPD, heart failure and dementia enrolled in a structured palliative care home-based program.[2] The Italian study found a dose response effect, where two or more home care visits per week was required to see a reduction in prolonged hospital stays[7] while participants in the US study followed a structured approach and received four to six home visits per week by nurses and one to three visits by social workers after enrolment followed by a less intense maintenance phase. [2] We did not have data on the intensity of community-based palliative care for this study apart from duration of enrolment in the service. Further work is required to determine whether the effect on length of hospital stay in non-cancer decedents is offset by the observed reduction in the rate of hospital admissions or whether the form and intensity of palliative care service delivered to decedents with non-cancer conditions can be improved.

The strengths of this study are the large population base and wide inclusion criteria resulting in a broad generalisability, particularly from a provider and policy viewpoint. Our conclusions are widely generalizable in the Australian context and may also apply internationally where similar models of community-based palliative care are employed. Limitations include a lack of detail on the intensity of the community-based palliative care provided and reliance on a single community-based palliative care provider whose main service area was metropolitan. Decedents living in rural and remote areas could have had access to more ad hoc palliative type care not accounted for in this study. Some decedents could have accessed palliative care support through patient organisations such as the Motor Neurone Disease Association. Such potential misclassification would have reduced our statistical power to observe differences. Another limitation was the relatively small number of decedents with non-cancer conditions who received community-based palliative care. This reduced the power to detect differences. While access to specialist palliative care for non-cancer conditions has increased over a ten year period, there is obvious room for further improvement.[15] The lack of a date of diagnosis, data on disease severity and duration was another study limitation.

We conclude that a reduced rate of hospital admissions and mean length of stay for people in the last year of life was associated with periods of time they were enrolled in a community-based palliative care service. The reduction in rate of admissions was evident, on average, five months before death and even earlier for older decedents. The association of community-based palliative care with a reduction in length of stay was more variable with every underlying cause of death showing different patterns over time.
